# Drug-Induced Gum Overgrowth With Low-Dose Amlodipine: A Case Report

**DOI:** 10.7759/cureus.25220

**Published:** 2022-05-22

**Authors:** Sadikshya Bhandari, Shisir Siwakoti, Shreeya Shrestha, Kushal Gautam, Samikshya Bhandari

**Affiliations:** 1 Internal Medicine, Dhulikhel Hospital, Dhulikhel, NPL; 2 Internal Medicine, Paediatric Research Unit, Patan Academy of Health Sciences, Lalitpur, NPL; 3 Internal Medicine, Nepal Medical College Teaching Hospital, Kathmandu, NPL

**Keywords:** dental health, digo, gum overgrowth, gum hypertrophy, adverse effects, antihypertensive drugs, amlodipine

## Abstract

Drug-induced gingival overgrowth is an adverse effect of certain drugs, including amlodipine, in genetically susceptible individuals. Although the exact mechanism of gingival hypertrophy remains unclear, a unifying multifactorial hypothesis has been constructed. Gingival hypertrophy causes difficulty in speech and mastication, poor oral hygiene, and poor aesthetic appearance. Here, we present the case of a 49-year-old woman who developed gum hypertrophy following amlodipine use for two years. Maintenance of oral hygiene and substitution of offending agent is commonly the first step in management.

## Introduction

Drug-induced gingival overgrowth (DIGO) is a commonly seen adverse effect of certain drugs administered for non-dental use such as anticonvulsants (especially phenytoin), immunosuppressants (mainly cyclosporine), and calcium channel blockers (mainly nifedipine) [1]. These drugs affect the periodontium, mainly the gingival connective tissue, with similar mechanisms, resulting in similar clinical and histological findings [2]. Here, we present a case of a 49-year-old woman with hypertension who developed DIGO secondary to amlodipine use.

## Case presentation

A 49-year-old perimenopausal woman presented to the outpatient clinic with a one-year history of swollen gums. The swelling was continuously present and slowly progressive. The patient had pain in her teeth and gums and had a foul-smelling watery discharge from her gums; moreover, food intake caused mild bleeding. She also had mild bilateral pitting pedal edema, with no skin changes. Her last menstrual period was two months back, and her cycles have been irregular, with bleeding lasting up to 10-12 days since the previous year. The patient had been taking 5 mg amlodipine of the same brand for three years for hypertension. She did not report having similar symptoms previously, and there was no past or current history of other drug intake. There was no history of similar symptoms in her family.

**Figure 1:**
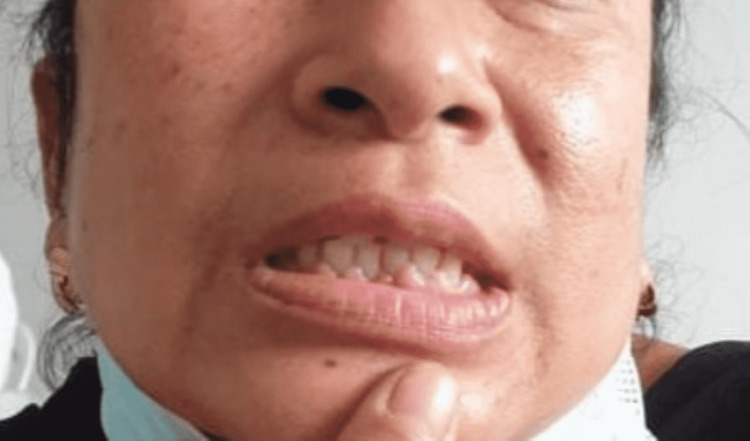
Diffuse gingival enlargement involving marginal, attached, and interdental gingiva on the buccal side mandibular anterior teeth.

As seen in Figure 1, intraoral examination revealed diffuse gingival enlargement involving marginal, attached, and interdental gingiva on both the buccal and lingual/palatal sides of the maxillary and mandibular anterior teeth, with mandibular gingiva exhibiting more severe changes. The patient’s oral hygiene was fair. On palpation, the enlarged gingiva was firm, fibrotic, and nontender.

She had initially consulted the periodontics department, where a provisional diagnosis of amlodipine-induced gum enlargement superimposed with inflammation was made based on clinical evaluation. She underwent mechanical and chemical plaque and calculus removal and received instructions on oral hygiene maintenance. She was then referred to the internal medicine outpatient department.

The patient was scheduled for gingivectomy. Preoperative blood biochemistry analysis revealed increased fasting blood glucose and triglyceride levels. All other parameters assessed were within normal limits (Table 1). Gingivectomy was performed, and a tissue specimen was sent for histopathological examination.

**Table 1:** Pre-gingivectomy investigation findings. HDL: high-density lipoprotein; LDL: low-density lipoprotein

Parameters	Findings	Normal value
Total leukocyte count (/µL)	6500	4000–11000
Neutrophil %	57	40–60
Lymphocytes %	35	20–40
Monocytes %	6	2–8
Eosinophils %	2	0–6
Hemoglobin (g/dL)	14	11.5–15
Platelets (/µL)	365,000	150,000–450,000
Sodium (mmol/L)	141	135–145
Potassium (mmol/L)	3.7	3.7–4.7
Urea (mg/dL)	23	14–23
Fasting blood sugar (mmol/L)	154	<100
Total cholesterol (mg/dL)	113	<200
HDL cholesterol (mg/dL)	45	>60.0
LDL cholesterol (mg/dL)	61	<100
Triacylglycerol (mg/dL)	180	<150

**Figure 2:**
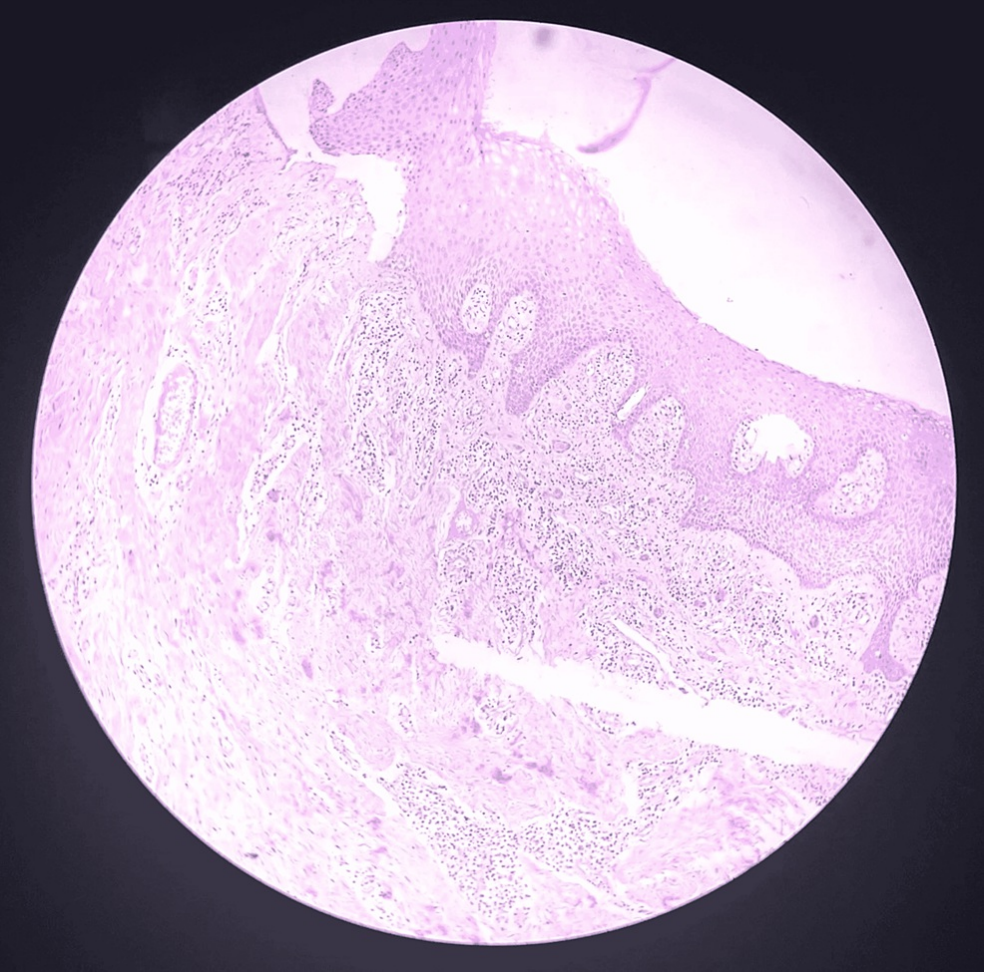
Gingival section showing keratinized stratified squamous epithelium overlying the fibrocellular connective tissue (Hematoxylin & Eosin, x10 magnification).

**Figure 3:**
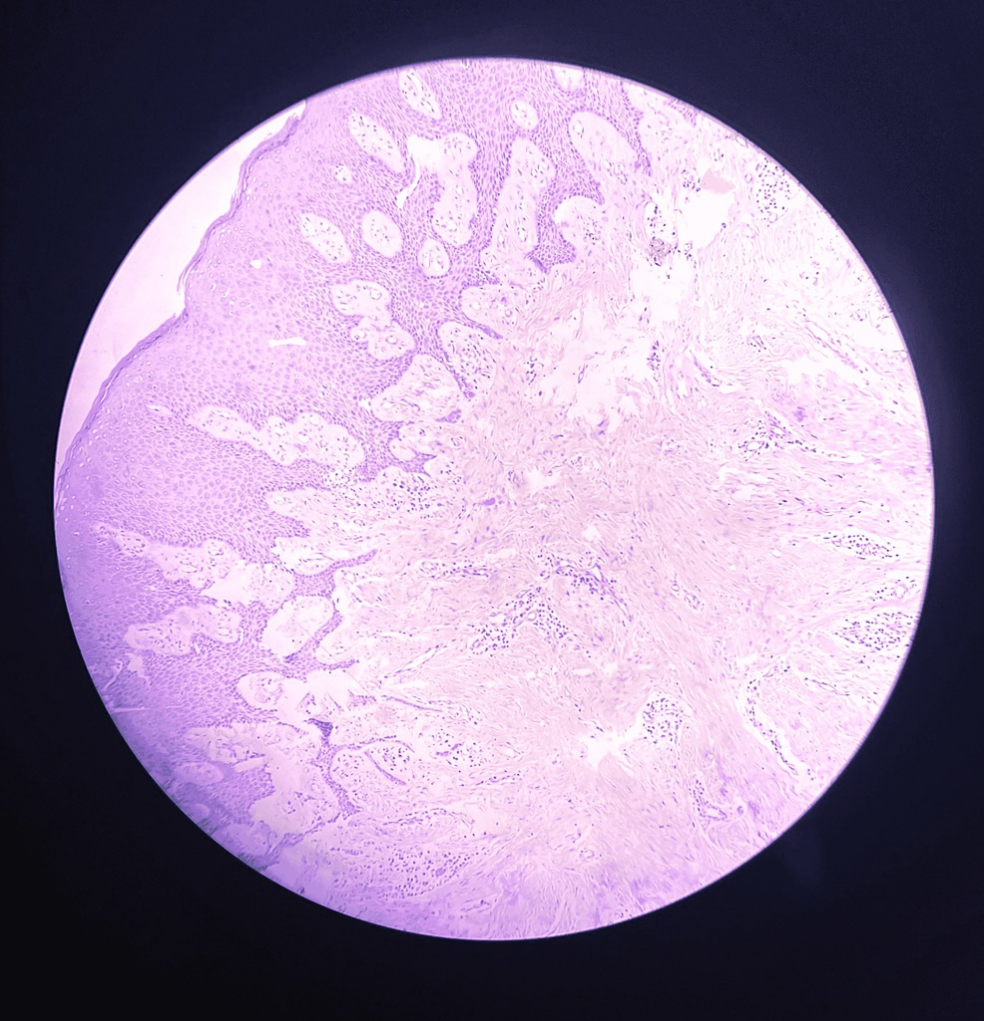
Gingival section showing loss of keratinization and inflammatory cell infiltration corresponding to the site of inflammation (Hematoxylin & Eosin, x10 magnification)

**Figure 4:**
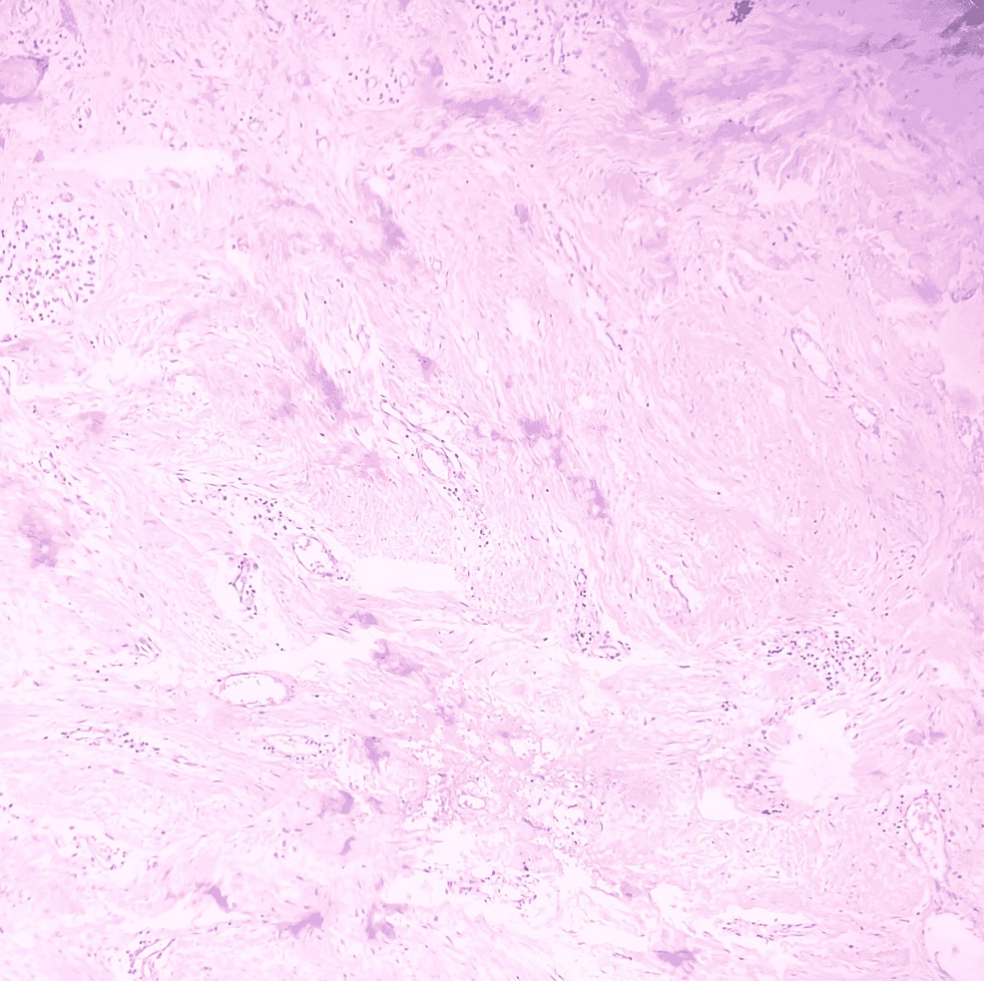
Gingival section showing dense bundles of collagen fibers, fibroblasts, endothelial cells, and few blood vessels engorged with RBCs and inflammatory cells (Hematoxylin & Eosin, x40 magnification)

As seen in Figure 2, Figure 3, and Figure 4, histopathological examination revealed keratinized stratified squamous epithelium overlying the fibrocellular connective tissue, with dense bundles of collagen fibers, fibroblasts, endothelial cells, and a few blood vessels engorged with red blood cells and inflammatory cells. 

**Figure 5:**
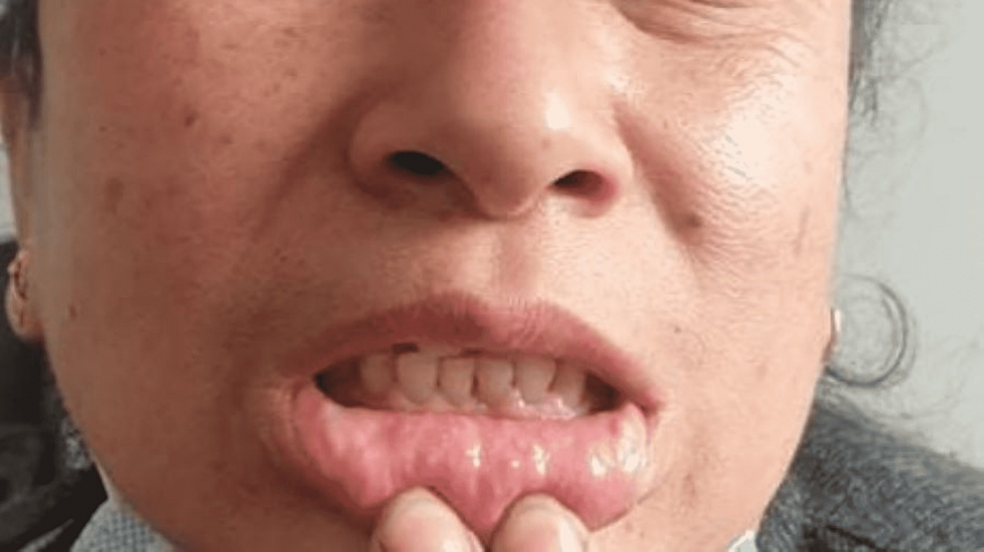
Slight enlargement of the gingiva on the anterior mandibular teeth. Significant improvement after one month of discontinuing amlodipine.

The diagnosis of drug-induced gingival enlargement was confirmed, and the patient was advised to change her antihypertensive medication from amlodipine 5 mg to losartan 50 mg. At the one-month follow-up after switching to losartan, the patient had significantly reduced swelling as seen in Figure 5 and no pain, discharge, or bleeding.

## Discussion

Amlodipine is a dihydropyridine calcium channel blocker with structural similarities to nifedipine, which is a common cause of gum hypertrophy [3]. Significant gingival overgrowth (SGO) is observed in 6.3% and 1.7% of people taking nifedipine and amlodipine, respectively [3], with a male preponderance [3,4]. Amlodipine-induced gingival overgrowth is generally reported at the dose of 10 mg/day within three months of drug initiation [5,6]. In our patient, however, the enlargement was noticed after two years of taking the drug at 5 mg/day.

Seymour et al. postulated that DIGO may have a genetic predisposition, given that among people using the same drug in the same amount or frequency, only some develop DIGO; moreover, the severity of gingival overgrowth is different in different people [6]. Although different genotypes have been postulated, the exact genetic association has not been determined [6,7]. An association of gingival hyperplasia induced by calcium antagonists with genetic MDR1 polymorphism is also suggested [8]. In another study done in 1996 in post-transplant patients, Thomason et al. determined that patients with HLA-B37 are likely to be protected from gum hypertrophy following cyclosporine use and so experience considerably less severe overgrowth than those who do not express this allele. However, as this allele is only expressed by 4% of patients without clinically significant overgrowth, its effect seems limited [7]. However, it has brought forward the concept of individualized treatment in patients according to their HLA genotype. 

Multiple etiopathogenetic mechanisms have been proposed for DIGO; however, the exact cause remains unclear. Because numerous drugs with variable pharmacodynamics can cause DIGO, it may have a multifactorial pathogenesis [9]. A unifying hypothesis states that anticonvulsants, immunosuppressants, and calcium channel blockers all cause cation flux inhibition [2]. A 2013 study reported that antiepileptic drugs block sodium channels, thus decreasing the action potential amplitude, which results in reduced calcium entry activation of potassium channels [10]. Decreased cation influx of folic acid active transport within gingival fibroblasts leads to decreased cellular folate uptake, which decreases the synthesis and activation of matrix metalloproteinases, a group of enzymes responsible for the breakdown of collagen. This reduces collagenase activity, causing decreased degradation and, thus, connective tissue accumulation, eventually presenting as DIGO [9,11].

Periodontal hygiene appears to play a major role in DIGO. Bacterial plaques allow the concentration of drugs in the area of buildup and produce an inflammatory state, leading to increased fibroblast proliferation, which assists in DIGO formation [9]. The severity of gingival overgrowth is directly proportional to the degree of plaque buildup and plaque-induced inflammation [12].

Many studies have demonstrated a drug-induced increase in glycosaminoglycans or connective tissue production secondary to gingival fibroblast proliferation. Most of the increase in connective tissue is mediated by inflammatory cytokines, including interleukin-1 beta, interleukin-6, interleukin-8, tumor growth factor beta1, and prostaglandin E2, which are secreted as part of an inflammatory response to the drugs and lead to fibroblast overgrowth [2,9]. A study hypothesized that inflammation is a prerequisite for the development of DIGO because, in the non-inflamed gingiva, fibroblasts were less active or quiescent even in the presence of drugs [13].

The first step in the management of DIGO is to stop the offending drug and replace it with an alternative after a physician consultation. Good oral hygiene practices should be followed, including plaque cleaning by dental professionals. For persistent cases, gingivectomy with or without flap surgery is warranted [14]. In our patient, gingivectomy was performed to control the plaque, and histological examination revealed hyperplastic epithelium and underlying connective tissue with dense collagen fibers, fibroblasts, proliferating endothelial cells, and blood vessels engorged with red blood cells and intense inflammatory cells, comprising mainly plasma cells; these features are characteristics of hyperplastic gingiva [15].

Similar case reports of gum hypertrophy have been published following amlodipine use in hypertension. In Table 2, a comparison of the basic parameters has been made between the different cases.

**Table 2:** Comparison of the indexed case with similar published cases. NA: Not Available

Parameters	Current study	Tripathi et al., 2015 [5]	Portnoy et al., 2022 [16]	Misra et al., 2021 [17]	Joshi et al., 2013 [18]
Age (Years)/Gender	41/F	50-65/NA	52/M	67/ F	45/M
Amlodipine dose (mg)	5	5	10	5	5
Duration of use (Years)	2	3	7	NA*	0.5
Comorbidities	Hypertension	Hypertension	Left ventricular hypertrophy, prediabetes, chronic right shoulder pain, obesity	Hypertension	Hypertension
Treatment (Substitution for amlodipine was done in every case)	Plaque removal + Gingivectomy + Oral prophylaxis	Internal bevel gingivectomy and flap operation + Oral prophylaxis	Oral Prophylaxis + (Planning for teeth extraction)	Lost to follow up.	Oral prophylaxis tooth scaling + Root planing
Resolved in (months)	12	NA	NA	NA	1.5

## Conclusions

We presented a case of DIGO associated with low-dose amlodipine causing difficulties in mastication, plaque buildup, and poor aesthetic appearance. The patient underwent a gingivectomy and amlodipine was replaced with losartan, which swiftly and markedly reduced the symptoms. She was provided instructions on good oral hygiene to prevent future recurrences. For more persistent cases, surgical management is recommended. 
